# Supplementary material for: Effects of long working hours on metabolic dysfunction-associated steatotic liver disease, with and without increased alcohol intake, in healthy workers: A 10-year cohort study
Source: PLoS One. 2025 Nov 6;20(11):e0336569. doi: 10.1371/journal.pone.0336569 (PMC12591466; doi:10.1371/journal.pone.0336569)
Supplement: S1 Table — (DOCX) [file pone.0336569.s001.docx]

**Supplementary Table S1. Temporal distribution of participant exclusions at each stage of study population selection**

| **Baseline period** | **Participants at baseline, n** | **Excluded at stage 1, n(%)** | **Excluded at stage 2, n(%)** | **Excluded at stage 3, n(%)** | **Excluded at stage 4, n(%)** | **Remaining participants, n** |
| --- | --- | --- | --- | --- | --- | --- |
| 2012-2014 | 248755 | 133 (0.05) | 163830 (65.9) | 4617 (5.45) | 24245 (30.24) | 55930 |
| 2015-2017 | 103980 | 68 (0.07) | 65581 (63.11) | 1562 (4.08) | 7843 (21.33) | 28926 |
| 2018-2020 | 83175 | 42 (0.05) | 51632 (62.11) | 1262 (4.01) | 5843 (19.32) | 24396 |
| 2021-2023 | 31078 | 21 (0.07) | 19683 (63.38) | 441 (3.88) | 2831 (25.89) | 8102 |
| Total, n | 466988 | 264 | 300726 | 7882 | 40762 | 117354 |
