# Supplementary material for: Effects of long working hours on metabolic dysfunction-associated steatotic liver disease, with and without increased alcohol intake, in healthy workers: A 10-year cohort study
Source: PLoS One. 2025 Nov 6;20(11):e0336569. doi: 10.1371/journal.pone.0336569 (PMC12591466; doi:10.1371/journal.pone.0336569)
Supplement: S2 Table — (DOCX) [file pone.0336569.s002.docx]

**Supplementary Table S2. Subgroup analyses of the association between long working hours and the risk of MASLD with Bonferroni-adjusted p-values**

| **Subgroup** | **HR (95% CI)** | **p** | **p_bonf** |
| --- | --- | --- | --- |
| Women | 1.06 (0.81 – 1.37) | 0.6747 | 1 |
| Men | 1.14 (0.99 – 1.32) | 0.0714 | 0.2856 |
| Aged ≤47 years | 1.20 (1.05 – 1.38) | 0.0078 | 0.0313 |
| Aged ≥48 years | 1.04 (0.75 – 1.45) | 0.8087 | 1 |
| HR, hazard ratio; CI, confidence interval; | |  |  |

*p_bonf*: p-value adjusted using the Bonferroni correction for multiple subgroup comparisons.
