# Supplementary material for: Effects of long working hours on metabolic dysfunction-associated steatotic liver disease, with and without increased alcohol intake, in healthy workers: A 10-year cohort study
Source: PLoS One. 2025 Nov 6;20(11):e0336569. doi: 10.1371/journal.pone.0336569 (PMC12591466; doi:10.1371/journal.pone.0336569)
Supplement: Attachment — Submitted filename: Response to the Editor and reviewers.docx [file pone.0336569.s004.docx]

**Response to the Academic Editor**

**1. Journal Style Requirements**
*Editor’s comment:* When submitting your revision, we need you to address these additional requirements.
*Response:* We have revised the manuscript to comply with the PLOS ONE formatting guidelines. Headings and structure have been modified according to the required style.

**2. Funding Statement**
*Editor’s comment:* Please provide an amended statement that declares all sources of support, and include the sentence “There was no additional external funding received for this study.”
*Response:* We have revised the Funding Statement as requested. The sentence *“There was no additional external funding received for this study.”* has been included in the cover letter and the Funding Statement has been updated accordingly.

**3. Data Sharing**
*Editor’s comment:* Please clarify the reasons for data restrictions and provide details regarding conditions for access.
*Response:* We have updated the Data Availability statement to clearly explain the reasons why the data cannot be publicly shared and to specify the conditions under which de-identified data may be accessed. The revised statement has also been included in the Acknowledgments section of the manuscript.

**4. Ethics Statement**
*Editor’s comment:* The ethics statement should appear only in the Methods section.
*Response:* The ethics statement is now included exclusively in the Methods section, and duplicate statements in other sections have been removed.

**5. Reference Marked as “Unpublished”**
*Editor’s comment:* The reference (Kivimäki et al.) has currently not yet been accepted for publication. Please remove it from the References and cite it in the text as “Unpublished.”
*Response:* We would like to clarify that according to PubMed, the following article is indexed with publication details:
*Kivimäki M, Virtanen M, Kawachi I, Nyberg ST, Alfredsson L, Batty GD, et al. Long working hours, socioeconomic status, and the risk of incident type 2 diabetes: a meta-analysis of published and unpublished data from 222,120 individuals. Lancet Diabetes Endocrinol. 2015 Jan;3(1):27–34. Epub 2014 Sep 25. doi: 10.1016/S2213-8587(14)70178-0.*

Since this article appears in PubMed with a DOI and full citation, we would appreciate the Editor’s guidance on why it should be considered “unpublished.”

**Response to Reviewer #1**

We thank the reviewer for the positive assessment of our manuscript and for the constructive suggestions that helped us improve clarity, rigor, and consistency. We address each point below and indicate where changes have been made in the revised manuscript.

**Comment 1**

*The article does not sufficiently establish the origins of the term "metabolic dysfunction-associated steatohepatopathy (MASLD)" in the Introduction and Methods. It is crucial to reference Rinella et al. (2023), which is the inaugural international Delphi Consensus that established the terminology and diagnostic criteria for MASLD, replacing the former NAFLD classification.*

**Response:**
We thank the reviewer for this valuable suggestion. We have revised the Introduction to clearly trace the origins of the MASLD terminology and diagnostic framework to the 2023 international multisociety Delphi consensus (Rinella et al., 2023). We now emphasize that the consensus replaced the former NAFLD classification and established MASLD as the preferred term to highlight the cardiometabolic risk factors associated with steatotic liver disease. The text has been revised accordingly (Introduction, lines 36–44).

**Comment 2**

*The mechanism section relies on prior studies without introducing novel pathways; underscore the clinical significance of the MASLD classification in the Discussion section.*

**Response:**
We agree with the reviewer’s comment. To address this, we have added a new paragraph in the Discussion emphasizing the clinical significance of the MASLD classification. Specifically, we note that by requiring both hepatic steatosis and at least one cardiometabolic risk factor, MASLD provides a more clinically relevant framework than NAFLD. This definition allows for earlier identification of individuals at risk for cardiovascular and metabolic diseases, improves risk stratification, and facilitates preventive interventions. It also aligns liver disease management with broader strategies in cardiometabolic medicine and public health. These revisions can be found in the Discussion (lines 291-296).

**Comment 3**

*The paper lacks a prospective efficacy analysis, and the Cox model was validated but lacked multiple comparison corrections; a Bonferroni correction is advised.*

**Response:**
We appreciate the reviewer’s concern. In response to the reviewer’s recommendation, we applied Bonferroni correction to the subgroup analyses (sex and age strata, n=4). The association between long working hours and incident MASLD in participants aged ≤47 years remained statistically significant after correction (HR 1.20, 95% CI 1.05–1.38; adjusted p=0.031), whereas other subgroup analyses did not remain significant. These results have been added as Supplementary Table S2, and the corresponding text has been updated in the Methods, Results and Discussion sections (lines 170-171, 223-226, 254-257).

**Comment 4**

*The flowchart should label the temporal distribution of excluded cases to check for selection bias. Table headings were misplaced (P5 Table1 should be above the table); reference 17 (Ryu et al. 2015) was not cited in the text; and the abbreviation MASLD was not initially defined. A thorough review is recommended for the next manuscript to prevent these errors.*

**Response:**
We appreciate the reviewer’s careful assessment and have addressed each point as follows.

1. **Flowchart (temporal distribution of exclusions).**
   Although temporal labeling is not routinely requested in flowcharts, we agree that providing this information improves transparency regarding potential selection bias. We therefore revised Figure 1 to indicate in the figure legend that exclusions occurred across the study period without clustering, and we added a new Supplementary Table S1 reporting the distribution of excluded participants by exclusion category across 3-year baseline periods. These additions allow readers to evaluate temporal patterns of exclusion. (Figure 1, legend; Supplementary Table S1).
2. **Table headings.**
   We appreciate the reviewer’s careful comments. We have double-checked the formatting of Table 1 in the submitted manuscript and confirmed that the table heading is appropriately placed above the table, in accordance with journal guidelines.
3. **Reference 17 (Ryu et al., 2015).**
   We thank the reviewer for the careful reading. Reference 17 (Ryu et al., 2015) is cited in the text (line 140). We have double-checked and confirmed that the citation is properly inserted.
4. **Abbreviation.**
   The abbreviation MASLD is now defined at first mention in both the Abstract and Introduction, and is used consistently thereafter throughout the manuscript.

**Response to Reviewer #2**

**1. Introduction and Background**

**Comment:** The introduction effectively sets the context with global and Korean-specific data on working hours. However, it could briefly elaborate on why MASLD is a priority outcome (e.g., its rising prevalence and links to cardiometabolic disease beyond NAFLD). Please add 1–2 sentences in lines 58–66 referencing the global burden of MASLD (e.g., cite a recent review on its epidemiology) to strengthen the rationale.

**Response:**
We thank the reviewer for this helpful suggestion. We have revised the Introduction (lines 45-48) to highlight why MASLD is a priority outcome by emphasizing its global burden, rising prevalence, and clinical significance beyond NAFLD. In particular, we added that MASLD affects nearly 38% of adults worldwide and is projected to exceed 55% by 2040, with cardiovascular disease being the leading cause of death in affected individuals. To strengthen the rationale, we have cited recent epidemiological reviews (Younossi et al., 2025).

**2. Methods**

**Comment 1:** In lines 118–128, please specify if overtime was explicitly included in the self-reported question or if it relies on participant interpretation.
**Response:**
We appreciate the reviewer’s comment. We clarify that overtime was explicitly included in the self-reported question on weekly working hours. The Methods section (lines 108-109) now reads: *“Working hours were assessed using the following question: ‘How many hours did you work in a week on average in your job for the past year, including overtime?’”*

**Comment 2:** For subgroup analyses (lines 174–177), it is better to justify the age cutoff (≤47 vs. ≥48 years) more explicitly—e.g., if it is based on the 90th percentile to capture younger vs. older workers.
**Response:**
We agree and have revised the Methods (lines 121-123) to clarify that the cutoff corresponded to the 90th percentile of age in the study population, allowing us to distinguish younger from older workers.

**Comment 3:** Please add a sentence on power calculations or sample size justification, given the low number of long-hour workers (n=2,243 vs. 115,111 in the reference group).
**Response:**
We thank the reviewer for this suggestion. Although the number of long-hour workers was relatively small compared to the reference group, the overall cohort size ensured adequate statistical power for subgroup analyses. We added a sentence in the Methods (lines 171-173) to clarify this.

**3. Results**

**Comment:** Please ensure consistency in reporting: The abstract says "higher risk of developing MASLD" (HR 1.17), but specify it's for ≥60 vs. 35–59 hours.
**Response:**
We agree and have revised the Abstract (line 11-13) to specify that the comparison was between participants working ≥60 hours per week and those working 35–59 hours per week.

**4. Discussion**

**Comment 1:** It is better to discuss policy implications more explicitly: Given Korea's work-hour reforms (Ref. 4), how might these findings inform thresholds like 52 or 60 hours/week?
**Response:**
We appreciate this valuable suggestion. We have expanded the Discussion (lines 352-357) to explicitly address the policy implications of our findings in light of Korea’s work-hour reforms, noting that our results support the importance of monitoring thresholds such as 52 and 60 hours per week.

**Comment 2:** Please add a brief note on why the association persisted in MASLD alone vs. MASLD+MetALD, perhaps tying it to alcohol's role in long-hour workers (higher heavy drinking at baseline).
**Response:**
We thank the reviewer for this insightful comment. We have added a brief note in the Discussion (lines 248-252) suggesting that differential alcohol consumption patterns among long-hour workers may partly explain the stronger association observed with MASLD alone compared with MASLD+MetALD.

**5. Generally**

**Comment 1:** Clarity and Writing: The manuscript is well-written overall, but some sentences are lengthy (e.g., lines 80–88). Break them for readability.
**Response:**
We agree with the reviewer’s comment. The long sentence in lines 80–88 has been revised and divided into shorter sentences to improve clarity and readability.

**Comment 2:** Dates: IRB approval and data access are dated 2025 (lines 93–97), while data collection ends in 2023. If this is a projection or typo, clarify; otherwise, there is no issue given the query date (Sep 2025).
**Response:**
We appreciate the reviewer’s careful attention. The Kangbuk Samsung Health Study is an ongoing cohort with continuous data collection. The Cohort Data Center periodically organizes the accumulated data and releases datasets to authorized researchers. For this study, data collected up to 2023 were available, while 2024 data are currently under processing. We clarified this in the Methods section (lines 65-67), noting that IRB approval for the present analysis was obtained in 2025 after data linkage was completed.

**Response to Reviewer #3**

**Comment 1:**

THE TITLE: the cohort study was conducted on patients being diagnosed with MASLD (with no alcohol or light alcohol intake), or MetALD (metabolic and alcohol-157 associated liver disease). both terms must be emphasized in the title and using “metabolic dysfunction-associated steatotic liver disease” alone will be misleading, since it means MASLD only. I suggest the following title “Effects of long working hours on Steatotic Liver Disease (MASLD and MetALD) in healthy workers: A 10-year cohort study”

**Response:**

We thank the reviewer for the helpful suggestion regarding the title. To more accurately reflect the study outcomes, we have revised the title to: *“Effects of long working hours on metabolic dysfunction-associated steatotic liver disease, with and without increased alcohol intake, in healthy workers: A 10-year cohort study.”*

**Comment 2:**

ABSTRACT: Since MetALD was being examined in this decade cohort study, the abstract did not reveal any information regarding those examined patients, regarding the objectives, results and conclusion sentences. I believe the authors should re-evaluate their manuscript in each details to be comprehensively summarized in the abstract.

**Response:**
We thank the reviewer for this valuable comment. We agree that the Abstract should comprehensively reflect the outcomes examined in our study. We have revised the Abstract accordingly. Specifically, the Objectives now state that the study assessed the association of long working hours with steatotic liver disease including both MASLD and MASLD+MetALD. In the Results, we added the hazard ratios for MASLD+MetALD alongside those for MASLD. Finally, in the Conclusion, we clarified that prolonged working hours were associated with an increased risk of steatotic liver disease, including both MASLD and MASLD+MetALD, particularly among younger and male participants.

**Comment 3:**

INTRODUCTION: MetALD disease should not be mentioned as part of MASLD system, and must be extensively differentiated in between, through providing etiologic manifestations of each disease (cause, criteria, alcohol intake), and main levels of their pathophysiologic progression. As well, recently published articles correlating the MetALD with workload or long working hours will be highly recommended to cite. However, the objectives of the study must reflect the conducted work in this cohort study, and NOT depending on correlation of MASLD with work load only.

**Response:**

We thank the reviewer for this important comment. We agree that MASLD and MetALD should be clearly distinguished, as they represent distinct disease categories within the recent consensus framework. Accordingly, we revised the Introduction to better differentiate MASLD and MetALD. We also clarified in the Objective statement that our study investigated the association between long working hours and steatotic liver disease, including both MASLD and MASLD+MetALD, rather than MASLD alone.

**Comment 4:**

MATERIALS AND METHODS:
The study analyzed data from 117,354 participants, enhancing statistical power and generalizability within the Korean working population, and through applying rigorous exclusion criteria (i.e., excluding participants with confounding conditions (e.g., viral hepatitis, steatogenic medications)) has improved the internal validity. As well, depending on MASLD diagnosis via abdominal ultrasonography is more accurate than indirect indices like the Hepatic Steatosis Index.
• Sensitivity Analysis was performed in the study, through including models that account for time-varying exposure to test robustness. However, the authors should clarify if any of multi-pronged strategies being applied to enhance data accuracy and validity, such as workplace records (to obtain actual working hours from employer records), digital tools (e.g., swipe cards, login/logout systems to track work duration more accurately), Cross-Validation, or Repeated Measures (Collect working hours data at multiple time points to identify inconsistencies or trends).
• Moreover, limited occupational detail (e.g., white-collar vs. blue-collar), which could influence exposure and outcomes, exclusion of changing work hours during follow-up in the main analysis, potentially omitting relevant variability. In addition, behavioral factors like sleep and diet were discussed but not measured, limiting mechanistic insights.

**Response:**

We thank the reviewer for recognizing the methodological strengths of our study, including the large sample size, rigorous exclusion criteria, and imaging-based diagnosis of MASLD. We also appreciate the constructive comments on data accuracy and measurement strategies. In our study, working hours were collected through standardized self-administered questionnaires, and objective workplace records or digital monitoring tools were not available. To minimize potential misclassification, we conducted a sensitivity analysis using repeated measurements and time-varying exposure models, which showed results consistent with the main findings. Importantly, in this time-dependent analysis we did not exclude participants whose working hours changed during follow-up, thereby capturing the variability in exposure that the reviewer highlighted. We agree that the lack of detailed occupational categories (e.g., white-collar vs. blue-collar) and the exclusion of such variability in the main analysis may still limit generalizability. Furthermore, behavioral factors such as sleep and diet were not measured, which we acknowledge as an important limitation that restricts mechanistic interpretation. These points have been clarified in the Methods and discussed as limitations.

**Comment 5:**

RESULTS:
Using Cox proportional hazards models with multiple adjustments (age, sex, lifestyle, socioeconomic factors) have strengthened the findings, and subgroup analyses, through stratification by age and sex have provided nuanced insights into vulnerable populations in the detailed tables
• The authors should further discussed any possible residual confounding lead to the modest association of MASLD with long work hours (adjusted HR for MASLD was 1.17), and the argument regarding the bias being developed from the exclusion of participants with missing data was inadequate and need in-depth justification.
• While the sensitivity analysis used three categories of working hours, the main analysis used only two, limiting granularity. Besides, the models did not test for interaction effects (e.g., between sex and working hours), which could reveal synergistic risks.

**Response:**

We thank the reviewer for these constructive suggestions. We agree that residual confounding may have contributed to the modest association observed between long working hours and MASLD (adjusted HR 1.17). Although we adjusted for major demographic, lifestyle, and socioeconomic factors, unmeasured variables such as sleep, diet, or occupational characteristics could not be accounted for, and this limitation has been further discussed. Regarding potential bias from excluding participants with missing data, we note that the Kangbuk Samsung Health Study is based on standardized comprehensive health screening examinations, in which the majority of participants provide complete questionnaire data and undergo abdominal ultrasonography at nearly every visit. As a result, missing data were minimal, which we consider a strength of this cohort.

With respect to working-hour categories, the main analysis used two groups (35–59 vs. ≥60 hours/week). This binary classification was selected to highlight the health impact of long working hours in survival analysis, and we have added this clarification in the Methods (Line 121-125). In addition, we conducted a sensitivity analysis using three categories (≤40, 41–52, and ≥53 hours/week), and the results were consistent with those of the main analysis, thereby supporting the robustness of our findings.

Furthermore, in response to comments from another reviewer, we applied Bonferroni correction in subgroup analyses to account for multiple testing, and the corrected p-values are presented in Supplementary Table S2.

Finally, while subgroup analyses suggested stronger associations in men and younger participants, we did not formally test statistical interaction terms in the Cox models. We believe that investigating potential effect modification by sex or age would be an important next step, and we plan to address this in future studies focusing on differential impacts of long working hours on MASLD across subgroups.

**Comment 5:**

DISCUSSION: The authors provide a comprehensive explanation of physiological pathways linking long working hours to MASLD, and findings are contextualized with previous studies, including cross-sectional and meta-analytic evidence. • Efforts must be paid to justify the absence of deep analysis of female-specific factors (e.g., unpaid labor, hormonal changes), while male vulnerability is well-discussed. Some proposed biological mechanisms (e.g., gut-liver axis disruption) are hypothetical and not directly measured, and further details regarding any unpublished analysis should be explained. In addition, the discussion lacks concrete suggestions for workplace or policy interventions.

**Response:**

We appreciate these insightful comments. First, we acknowledge that our dataset did not include variables on unpaid labor, reproductive or hormonal factors, or other female-specific exposures, which limited our ability to explore mechanisms underlying sex differences in depth. We have clarified this in the Discussion and emphasized the need for future studies to address these aspects (Line 333-337).

Second, we agree that certain biological mechanisms such as disruption of the gut–liver axis are hypothetical and were not directly measured in our study. We have revised the Discussion to explicitly note this point (Line 281-283).

Third, no unpublished analyses were conducted beyond what has been reported in the manuscript.

Finally, we have expanded the Discussion to include more concrete implications for workplace and policy interventions (Line 352-357).
